# Effect of an Electric Field on the Structure and Stability of Atmospheric Clusters

**DOI:** 10.1021/acs.jpca.3c07260

**Published:** 2024-01-13

**Authors:** Christopher David Daub, Theo Kurtén

**Affiliations:** Department of Chemistry, University of Helsinki, P.O. Box 55, Helsinki 00014, Finland

## Abstract

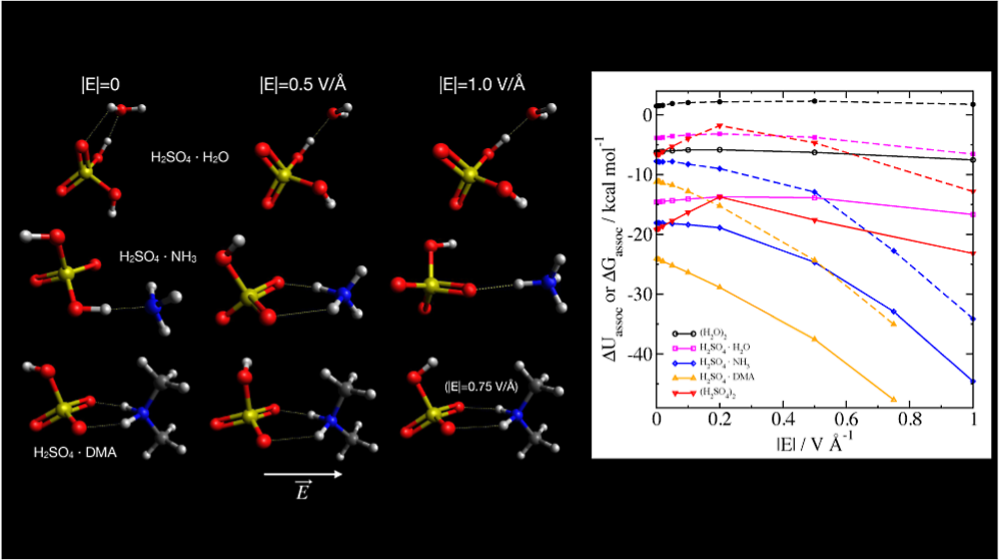

We study the influence of an applied electric field on the structure and stability of some common bimolecular clusters that are found in the atmosphere. These clusters play an important role in new particle formation (NPF). For low values of the electric field (i.e., |*E*| ≤ 0.01 V Å^–1^), we demonstrate that the field response of the clusters can be predicted from simply calculating the dipole moment of the cluster and the dipole moments of the constituent molecules and that the influence on the association energy of the cluster is minimal (i.e., <0.5 kcal mol^–1^). For higher field strengths |*E*| > 0.2 V Å^–1^, there can be more dramatic effects on both structure and energetics, as the induced dipole, charge transfer, and geometric distortion play a larger role. Although such large fields are not very relevant in the atmosphere, they do exist in some situations of experimental interest, such as near interfaces and in intense laser fields.

## 1 Introduction

The initial formation of acid–base clusters in the atmosphere represents an important step in new particle formation (NPF) that can lead to aerosol formation, which in turn can seed clouds. Despite the extensive work on these systems, according to the latest IPCC report, aerosol and cloud formation remain the largest uncertainty in estimating the future of terrestrial climate change.

Can electric fields affect the stability of acid–base clusters? In the atmosphere, field strengths |*E*| on the order of 10^5^ V m^–1^ = 10^–5^ V Å^–1^ are routinely produced due to the electric potential difference created between the ground and large thunderclouds.^1^ In some experimental cloud chambers used to study cluster formation, in particular the CERN CLOUD experiment, a sweeping field of 0.3 × 10^–5^ V Å^–1^ is used to remove charged particles from the chamber.^2^ The use of such a clearing field has been criticized by some who claim that the electric field could significantly affect the chemistry.^3^

Some experimental methods used to study atmospheric reactions, for example, chemical ionization mass spectrometry (CIMS)^4^ or electrospray ionization mass spectrometry (ESI-MS)^5^ also involve the use of electric fields to accelerate ions of interest. These field strengths are typically in the range of 1 × 10^–4^ V Å^–1^, with some experiments using larger fields (up to 1 × 10^–3^ V Å^–1^). Alterations in the electric field are known to affect the degree of ion fragmentation observed in some CIMS experiments, which is thought to be due to the field dependence of the collision energy of the ions.^6^

Even higher-field strengths may be relevant in some cases. For example, in ESI-MS, the actual electric field is highly nonuniform and may exceed 0.1 V Å^–1^ at the tip of the Taylor cone.^7,8^ Near gas–solid or gas–liquid interfaces, the presence of strong electric potential gradients also mean that large electric fields up to 0.1 V Å^–1^ may locally exist very close to the interface.^9^ Lasers are also a source of large electric fields on the order of 0.1 V Å^–1^, and these have recently been used to trap and levitate atmospheric aerosols.^10^

Simulations of bulk systems in electric fields, especially aqueous systems, using empirical force field models, have been a popular topic for many years; for some reviews, see e.g., refs (11) and (12). Computational studies of how electric fields affect small clusters, however, are less common. Various studies of small water clusters in an electric field have been done.^13−20^ Other interesting studies of clusters in electric fields include examinations of methanol clusters,^21^ cation–π interactions,^22^ Diels–Alder reactions,^23^ hydrated sodium and calcium ions,^24^ and the hydrated lithium cation.^25^ The presence of the electric field in molecular clusters generally introduces competition between more cyclic structures, maintaining a maximal number of hydrogen bonds, versus the electric field’s tendency to stabilize the alignment of molecular dipoles with the electric field, similar to what is seen in the formation of molecular water wires or water bridges, for example.^13,14,26^

Other than small water clusters, however, atmospheric clusters have not been examined to learn how they might be affected by an electric field. In this study, we applied an electric field of strength ranging from |*E*| = 0.005 to 1.0 V Å^–1^ (1 Å = 10^–10^ m) and used density functional theory (DFT) to examine how the field affects the structure and energetics of the water dimer, as well as bimolecular clusters of sulfuric acid (H_2_SO_4_) with water, ammonia, dimethylamine (DMA, (CH_3_)_2_NH), and the sulfuric acid dimer. These clusters represent the most important systems to consider in describing the initial stages of atmospheric NPF;^27−31^ for recent reviews of this topic, see e.g., Elm et al.^32^ and/or Engsvang et al.^33^

## 2 Methods and Models

All quantum chemical computations were done with the program ORCA, version 5.0.4.^34,35^ We used density functional theory with the ωB97X functional.^36−38^ Extensive previous work on acid–base clusters including sulfuric acid has shown this functional to be reliable.^28,29^ Geometry optimizations were completed using VeryTightSCF criteria on the self-consistent field part of the calculation, VeryTightOPT criteria on the geometry optimization, and the Defgrid3 keyword to define a very fine numerical grid.

In some previous works studying external electric fields in quantum chemical calculations,^25^ the field was approximated by placing fixed positive and negative charges equidistant from the system. In new versions of ORCA, it is possible to use an undocumented feature to apply the external field directly. We felt it prudent to ensure that the results using both methods were consistent. The results of these test calculations can be found in the Supporting Information (see Figure S1).

### 2.1 Field Response

We measured the field response of a system in an electric field by computing
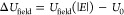
1where |*E*| is the magnitude of the electric field, and the subscript 0 denotes the value measured in the absence of the electric field. Note that we will always align the applied electric field vector  in the same direction as the system dipole moment  computed in zero field, so that the field effect is maximized. In defining the internal energy *U*, we included only the single-point electronic energy to make it easier to compare with results using other computational chemistry programs.

For estimating the effect of the electric field on atmospheric clusters, not only the electronic energy but also the Gibbs free energy *G* is relevant. Similar to eq 1, we can define
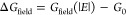
2where now *G* includes all thermal effects, zero-point energy, as well as the *TS* term computed from a vibrational frequency analysis. We used *T* = 298.15 K for all of the free-energy calculations in this work.

### 2.2 Association Energies

We also discuss the association energies, sometimes referred to as “binding” energies. Other similar terms can be used, such as dimer dissociation energy, etc., and care should be taken to precisely define what is being measured. Here, we define the field-dependent association energy as
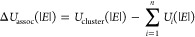
3where *U*_*i*_(|*E*|) is the field-dependent electronic energy of each individual molecule making up the cluster. Similarly, we can define
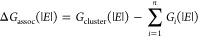
4

It is worth noting again here that each individual value of *U*(|*E*|) or *G*(|*E*|) comes from a separate optimization of each cluster or molecule. In other words, each molecule or cluster is in its minimum energy conformation, with the electric field optimally aligned with the dipole moment.

### 2.3 Association Energy of Ion Pairs in the Electric Field

The calculation of association energy described in Section 2.2 is appropriate for clusters of ions in the absence of an electric field or for clusters of neutral molecules with and without an electric field. However, for ionic clusters in an electric field, some other physics must be considered.

Consider a pair of oppositely charged point charges ±*q* in an electric field oriented in the same direction as the interparticle separation. The classical potential energy is given by
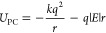
5where *k* = 8.99 × 10^9^ N m^2^ C^–2^ is Coulomb’s constant, *r* is the separation, and |*E*| is the magnitude of the electric field. At small *r*, the Coulombic attraction between the charges dominates. At larger *r*, the field dominates, causing the charges to separate. The point of balance between these effects, i.e., the location of the barrier for field-induced dissociation, can be found by computing
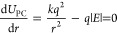
6Solving for *r*, we find that . Furthermore, the maximum value of *U*_PC_ at *r* = *r*_barrier_ is
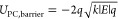
7

In a real chemical system, instead of *U*_PC_ → −∞ as *r* → 0, the repulsion between atoms at small separations would ensure that there will be a minimum value of *U*_PC_ = *U*_min_ at *r* = *r*_min_, where the system is at equilibrium in zero field (essentially, this amounts to introducing an infinite repulsive wall at *r* = *r*_min_). Therefore, we can approximate the difference in energy between the barrier and the minimum as another association energy Δ*U*_assoc,PC_ for two bound point charges, given by
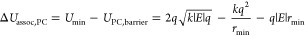
8

Depending on the system and the field strength, either the neutral dissociation pathway described by eq 3 or the ionic-field-driven dissociation described by eq 8 may be more energetically favorable.

### 2.4 Choice of Basis Set

When using ORCA, the external field effect cannot be included at the moment in analytical calculations of the gradient. This required us to use numerical methods to compute gradients necessary for geometry optimizations as well as vibrational frequencies necessary to compute the entropy. This increased the computational effort required considerably; please see the Supporting Information (Table S4) for some examples of the CPU hours required for numerical vibrational frequency calculations. To mitigate this added cost, we decided to use the unaugmented cc-pVTZ basis set instead of the aug-cc-pVTZ basis set^39^ used in most other work on sulfuric acid clusters.

Some test calculations with the aug-cc-pVTZ basis set were done to compare, and these results are included in the Supporting Information (Tables S2 and S3). By and large, the cc-pVTZ basis set does introduce a small error in that Δ*U*_assoc_ and Δ*G*_assoc_ are more negative compared with the aug-cc-pVTZ basis set by ∼1–2 kcal mol^–1^. This finding is consistent with what has been found by others^29^ in the zero-field case. In the absence of the electric field, this difference is almost the same as the basis-set superposition error (BSSE) computed with the counterpoise correction in the cc-pVTZ basis set. In high field strength, however, the BSSE in the cc-pVTZ basis set differs from the difference in energy between the cc-pVTZ and aug-cc-pVTZ results, suggesting that there can be some important contribution from the extra diffuse basis functions in the augmented basis set in high field strengths in some cases. Nevertheless, it seems that the qualitative effects of the electric field on the energetics and on the molecular structures are well described by the smaller cc-pVTZ basis set.

### 2.5 Choice of Dispersion Correction

It is well known that dispersion corrections are crucial to include in calculations involving noncovalent interactions such as molecular clusters. In this work, we consistently used Grimme’s D3 dispersion correction with zero damping.^40,41^ However, as a referee has pointed out, the implementation of dispersion corrections may make an exact comparison between different software packages difficult.

To investigate this possibility, we did some test calculations with Grimme’s D2 correction,^42^ which are reported in the Supporting Information (see Table S5). For the H_2_SO_4_·H_2_O cluster, we find the zero-field association energy Δ*U*_assoc_ = −14.6 kcal/mol with the D3 correction compared with −17.9 kcal/mol with the D2 correction. This difference is surprisingly large and certainly warrants careful checking to make sure dispersion corrections are accurately implemented and documented. Myllys et al.^29^ used Gaussian09 and its implementation of D3 to calculate Δ*U*_assoc_ = −14.3 kcal/mol using the same ωB97X functional and cc-pVTZ basis set as we used. It seems clear to us that the implementations of D3 in ORCA and in Gaussian09 are, if not exactly the same, producing very similar results.

## 3 Results

**Figure 1:**
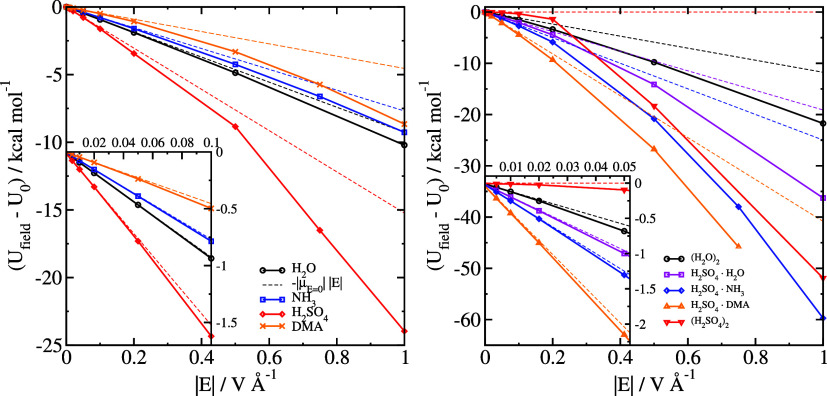
Difference in electronic energy between structures optimized in the electric field and without an electric field for single molecules (left) and clusters (right). The inset shows only the results at small field strengths. The thin dashed lines are estimates of the value of the energy of a dipole oriented in an electric field *E*, given by eq 9, where the value of the dipole moment μ is assumed to be equal to its value at zero field.

We begin by discussing the results of optimization of single molecules. In Figure 1, we show the difference in energies between structures optimized in the absence of an electric field and as a function of electric field. All of the raw data in this figure is available in Table S1 in the Supporting Information. For field strengths |*E*| ≤ 0.05 V Å^–1^, a simple model for the field response can be applied by calculating the dipole moment μ in the absence of an electric field and then approximating the response as
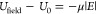
9

As the field strength is increased, due to nuclear and electronic rearrangements leading to an additional induced dipole, the field response becomes significantly larger than that predicted by eq 9. One way to improve the quantitative agreement at a higher field strength would be to also include the polarizability of the molecule/cluster. We have done so in the Supporting Information and show the results in Figures S2 and S3. It can also be seen from Table S1 that the effect of the electric field on the entropy and, hence, on the Gibbs free energy of individual molecules is minimal.

**Figure 2:**
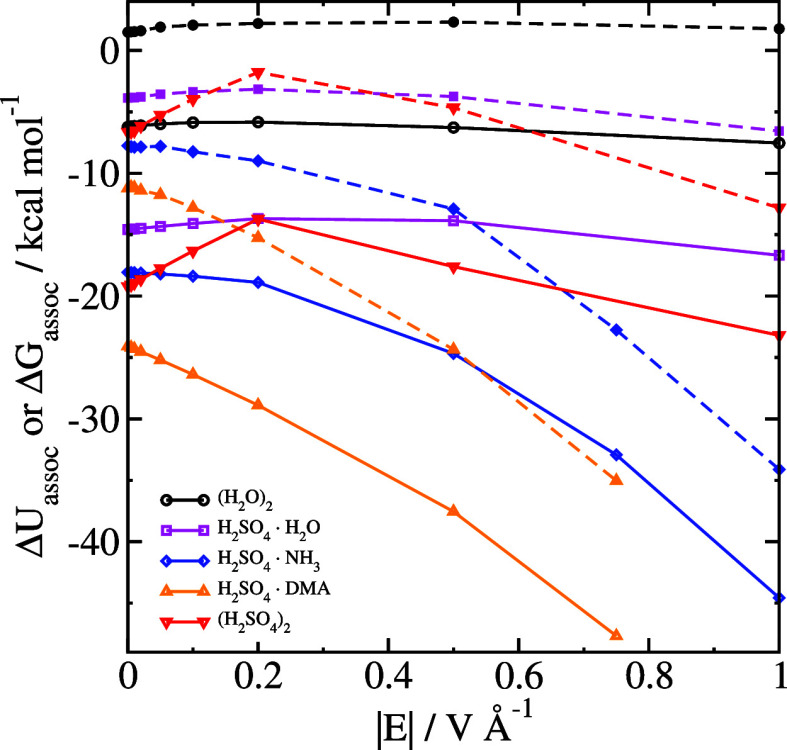
Association energies of clusters, either only the electronic energies (Δ*E*_assoc_, open symbols and solid lines) or the Gibbs free energy (Δ*G*_assoc_, closed symbols and dashed lines).

**Figure 3:**
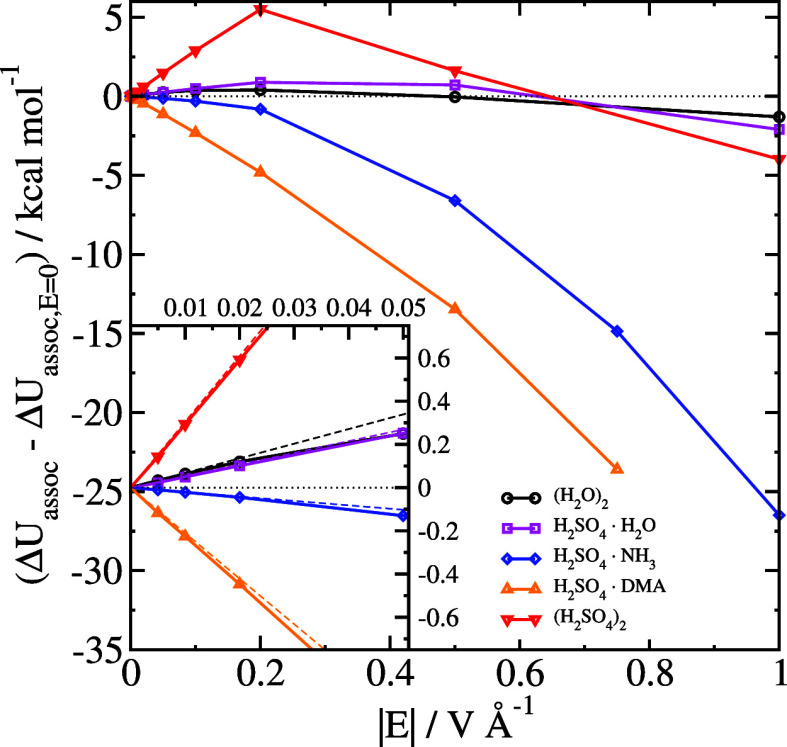
Difference in electronic association energy Δ*U*_assoc_ between structures optimized in an electric field and without an electric field. The inset shows only the results at small field strengths. The thin dashed lines are graphs of eq 10 for each system, with each of the dipole moments calculated in zero field.

In Figure 1, we also show the field response for clusters, and in Figures 2 and 3 we show our calculated values of Δ*U*_assoc_ and Δ*G*_assoc_. The raw data are tabulated in Table 1. Similar to single molecules, the field response in a low field can be approximated simply by eq 9, although the increased flexibility of the clusters means that deviations appear already for *E* > 0.02 V Å^–1^. Furthermore, as far as association energies Δ*U*_assoc_ are concerned, the difference between the value in the field and the value in the absence of the field can be approximated well by
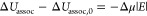
10where Δμ = μ_clust_ – , with μ_clust_ being the total dipole moment of the cluster and μ_*i*_ the dipole moment of each individual molecule in the cluster, all calculated in zero field.

**Table 1:** Electronic Energies *U*_field_ – *U*_0_, Dipole Moment μ, Entropy Term *TS*, Gibbs Free Energies *G*_field_ – *G*_0_, and Electronic Association Energies Δ*U*_assoc_ and Gibbs Free Energy for Association Δ*G*_assoc_ as a Function of the Electric Field Strength |*E*| for Clusters^a^

|*E*|/V Å^–1^	μ/D	*U*_field_ – *U*_0_/kcal mol^–1^	*S* × 298.15 K/kcal mol^–1^	*G*_field_ – *G*_0_/kcal mol^–1^	Δ*U*_assoc_/kcal mol^–1^	Δ*G*_assoc_/kcal mol^–1^
(H_2_O)_2_						
0	2.446	0	20.81	0	–6.236(−4.948)	1.486
0.005		–0.060	20.80	–0.060	–6.203	1.519
0.01		–0.122	20.81	–0.135	–6.172	1.534
0.02		–0.252	20.81	–0.258	–6.116	1.600
0.05		–0.681	20.65	–0.518	–5.986	1.901
0.1		–1.500	20.62	–1.304	–5.863	2.058
0.2		–3.379	20.52	–3.076	–5.833	2.201
0.5		–9.775	20.07	–8.951	–6.273	2.308
1.0		–21.727	19.53	–20.245	–7.541(−5.977)	1.765
H_2_SO_4_·H_2_O						
0	3.980	0	25.49	0	–14.578(−12.759)	–3.867
0.005		–0.100	25.44	–0.031	–14.555	–3.841
0.01		–0.196	25.48	–0.173	–14.529	–3.850
0.02		–0.392	25.46	–0.346	–14.477	–3.781
0.05		–0.998	25.41	–0.903	–14.327	–3.557
0.1		–2.069	25.41	–1.977	–14.090	–3.375
0.2		–4.450	25.49	–4.457	–13.693	–3.155
0.5		–14.128	25.73	–14.482	–13.863	–3.751
1.0		–36.271	25.34	–36.627	–16.677(−16.360)	–6.559
H_2_SO_4_·NH_3_						
0	5.201	0	25.91	0	–18.072(−16.545)	–7.756
0.005		–0.126	25.91	–0.129	–18.082	–7.837
0.01		–0.252	25.94	–0.297	–18.093	–7.880
0.02		–0.508	25.89	–0.498	–18.117	–7.858
0.05		–1.303	25.78	–1.175	–18.202	–7.807
0.1		–2.713	25.89	–2.789	–18.378	–8.252
0.2		–5.863	25.90	–6.072	–18.888	–8.993
0.5		–20.820	25.57	–19.601	–24.673	–12.910
0.75		–37.983	26.71	–37.692	–32.927	–22.755
1.0		–59.723	26.79	–59.158	–44.569(−42.400)	–34.115
H_2_SO_4_·DMA						
0	8.504	0	30.11	0	–24.072	–11.214
0.005		–0.217	29.85	0.060	–24.189	–11.123
0.01		–0.426	29.81	–0.097	–24.298	–11.173
0.02		–0.848	29.84	–0.512	–24.519	–11.395
0.05		–2.151	29.62	–1.497	–25.201	–11.746
0.1		–4.427	29.53	–3.577	–26.381	–12.795
0.2		–9.327	29.50	–8.326	–28.879	–15.244
0.5		–26.756	30.05	–26.057	–37.541	–24.345
0.75		–45.843	30.63	–45.629	–47.671	–35.029
(H_2_SO_4_)_2_, cfg. 1						
0	0.0	0	30.82	0	–19.219	–6.651
0.005		–0.011	30.80	0.013	–19.076	–6.616
0.01		–0.013	30.87	–0.076	–18.926	–6.532
0.02		–0.024	30.78	0.009	–18.628	–6.148
0.05		–0.096	30.83	–0.119	–17.746	–5.277
0.1		–0.353	30.84	–0.384	–16.330	–3.974
0.2		–1.392	31.07	–1.684	–13.723	–1.787
0.5		–18.334	29.83	–17.435	–17.605	–4.663
1.0		–51.904	31.86	–53.515	–23.200	–12.820

a All optimizations were performed as described in Section 2, starting from the global minimum geometry in zero field. Results in parentheses include BSSE.

It is worth noting that although eq 10 only fits the data at the smallest field strengths we have included in our calculations, these field strengths are still at least an order of magnitude larger than those measured in thunderclouds, or applied in experiments such as the CERN CLOUD chamber or the drift tube of a mass spectrometry apparatus.

The model represented by eq 10 is also a convenient way to rationalize the different results seen in different clusters. In some cases, in the low field, the association is less favorable since the dipole moment of the cluster is less than the sum of dipole moments of the molecules. As the field strength is increased more, the effects of charge transfer and geometric distortion lead to increasingly favorable association according to eq 3 in all of the systems we studied. However, at higher field strengths, ion pairs can also be dissociated by the electric field, as described in Section 2.3. Further details and analysis of what happens in each individual system will be provided in the following subsections.

### 3.1 H_2_O Dimer

Our results for the water dimer in the absence of an electric field agree well with previous theoretical and experimental results.^43−47^ As stated in Section 2, by using the cc-pVTZ basis set, we overestimated the association energy of the water dimer by ∼1 kcal mol^–1^ or so. Either inclusion of BSSE correction or the use of the aug-cc-pVTZ basis set is sufficient to bring our results closer to more rigorous computational determinations of the association energy, estimated to be −5.0 ± 0.1 kcal mol^–1^.^44^ Our value of Δ*G*_assoc_ = 1.5 kcal mol^–1^ is also in fair agreement with a more rigorous assessment, which gives a value of ∼1.8 kcal mol^–1^.^45^

When an electric field is applied to the water dimer, in the low field, we observed a small decrease in the magnitude of association energy. This decrease is well described by the simple model of eq 10. At increased field strength |*E*| > 0.2 V Å^–1^, the trend reverses, as charge transfer and geometric distortion play a larger role.

We can compare our results for the water dimer in an electric field with those of a few previous studies. Toledo et al.^16^ also reported a reduction in the magnitude of association energy of (H_2_O)_2_ in a low field. However, they do not observe the reversal of this trend in a higher field. This can be explained by the fact that they did not reoptimize the cluster geometry in the field, so that they would not have included the effect of molecular deformation caused by the field. Mondal et al.,^15^ by contrast, reported a small increase in the magnitude of the association energy as the field strength is increased, even in a low field. It seems unlikely that the good agreement we see between the simple models of eqs 9 and 10 and our data in a low field is erroneous. Rather, we suspect that the explicit calculation of the dimer dissociation energy using constrained optimization by Mondal et al. is incorrect by a small amount, perhaps due to some residual long-range interaction between the water molecules at the largest hydrogen bond separation they included (7 Å according to Figure 3 of ref (15)).

### 3.2 H_2_SO_4_·H_2_O Cluster

The H_2_SO_4_·H_2_O cluster in the absence of an electric field was well studied previously.^27−29,48^ We used the global minimum configuration from ref (27) as our starting point. In zero field, we got Δ*U*_assoc_ = −14.6 kcal/mol (−12.6 kcal/mol) and Δ*G*_assoc_ = −3.9 kcal/mol (−2.0 kcal/mol) using the cc-pVTZ (aug-cc-pVTZ) basis sets. For direct comparison between DFT-based calculations, Myllys et al. obtained Δ*U*_assoc_ = −14.3 and −12.4 kcal/mol using ωB97X-D3 with the cc-pVTZ and aug-cc-pVTZ basis sets, respectively,^29^ in good agreement with our results.

Currently, the most reliable theoretical calculations in the literature obtained Δ*G*_assoc_ = −2.6 kcal/mol based on coupled-cluster CCSD(T)-F12 calculations and a sophisticated treatment of how local and global anharmonicity affect the Gibbs free-energy calculation^48^ and Δ*G*_assoc_ = −2.0 kcal/mol based on ωB97X-D geometries and CCSD(T)-F12 single-point energy calculations extrapolated to the complete basis set (CBS) limit.^49^ There is also an experimental measurement of Δ*G*_assoc_ = −3.6 ± 1 kcal/mol,^50^ which the best theoretical determinations are in good agreement with. The high association free energy between H_2_SO_4_ and H_2_O, combined with the high atmospheric concentration of water vapor, supports the presence of high concentrations of the H_2_SO_4_·H_2_O cluster in the atmosphere.

Experimental results on Δ*U*_assoc_ are not available, nor were they given in the Partanen et al. benchmark study.^48^ The most reliable theoretical value we have been able to find is Δ*U*_assoc_ = −12.6 kcal/mol from ωB97X-D/aug-cc-pVTZ geometries supplemented with CCSD(T)-F12/CBS single-point energies,^49^ which is also in good agreement with Δ*U*_assoc_ = −13.1 kcal/mol obtained from a MP2/aug-cc-pV(D+d)Z optimization followed by MP4/aug-cc-pV(D+d)Z single-point energy calculation.^27^

**Figure 4:**
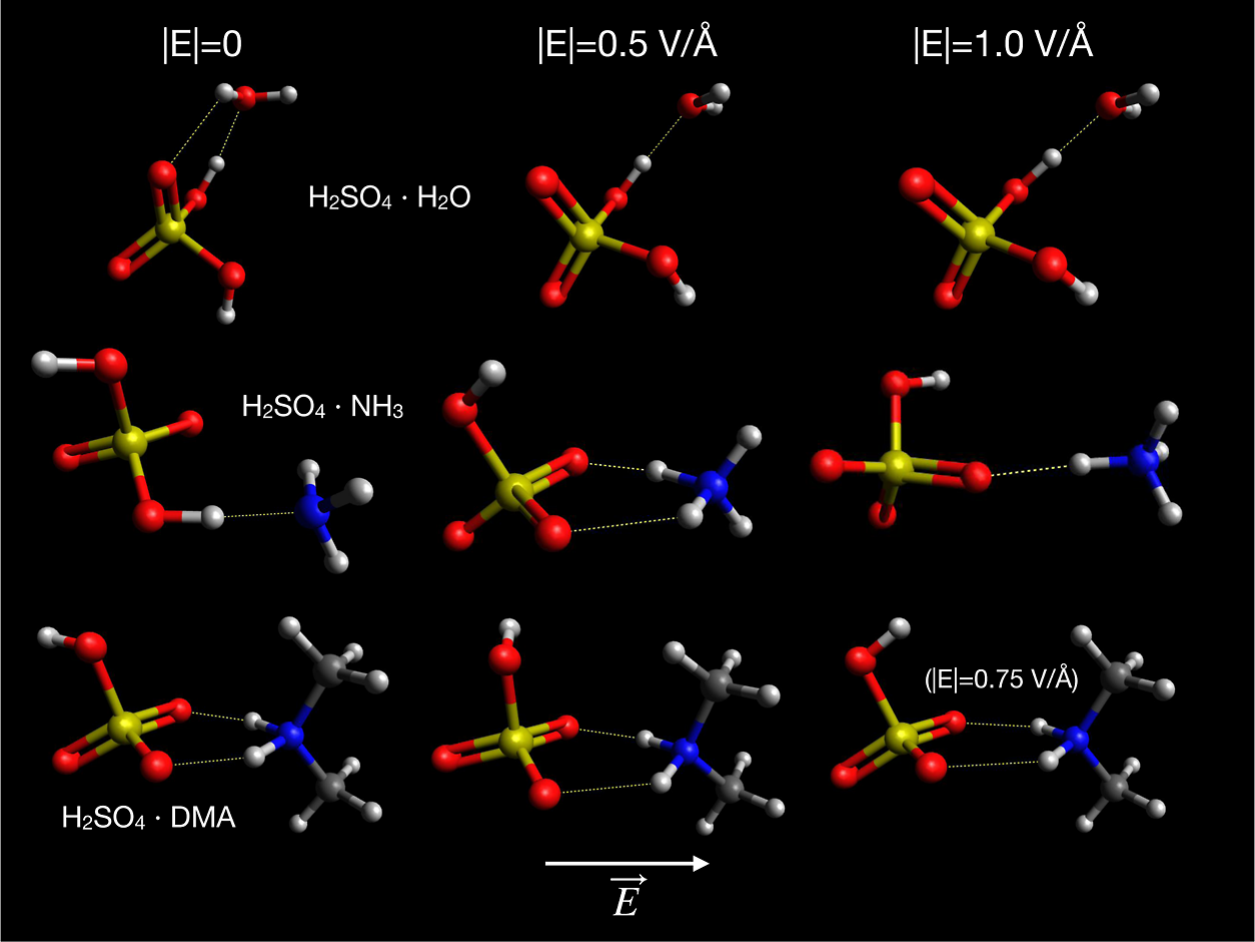
Optimized configurations of bimolecular clusters of H_2_SO_4_ with H_2_O, NH_3_, and DMA at different electric field strengths, with the field direction toward the right. Note that the high field configuration for H_2_SO_4_·DMA is at |*E*| = 0.75 V Å^–1^, while the others are at 1.0 V Å^–1^.

Similar to the case for the water dimer, the geometry of the hydrogen bond in this case has caused the dipole moment of the cluster to be less than the sum of dipole moments of individual molecules. As a result, the association energy at low-field strength is slightly lower than in the zero field case, until, at high field strength, molecular deformation and charge transfer combine to increase the association energy. Configurations of the H_2_SO_4_·H_2_O cluster optimized in zero field and in fields of 0.5 and 1.0 V Å^–1^ are shown in Figure 4. The geometry remains relatively unchanged even in a high field, with the main change being the breaking of one weak hydrogen bond as the water molecule’s dipole more strongly aligns with the field direction.

### 3.3 H_2_SO_4_·NH_3_ Cluster

In zero field, we obtained values of −18.1 and −7.8 kcal/mol for Δ*U*_assoc_ and Δ*G*_assoc_, respectively. This agrees well with Δ*U*_assoc_ = −17.9 and −16.5 kcal/mol with cc-pVTZ and aug-cc-pVTZ basis sets, respectively.^29^

Unlike the two previous cases, it can be seen from Figures 2 and 3 that the association energy of the sulfuric acid–ammonia system increases for all values of the field strength. Even in zero field, the optimal configuration of this cluster is such that the total dipole moment is greater than that of the sum of the monomer dipoles, indicating that the molecular dipoles are already well aligned and that there is already some amount of charge transfer.

Optimized configurations of the H_2_SO_4_·NH_3_ cluster at some different field strengths are shown in Figure 4. As the field strength is increased beyond 0.5 V Å^–1^, a proton transfer is induced from sulfuric acid to ammonia, producing a HSO_4_^–^·NH_4_^+^ ion pair. The electrostatic interaction between these charges leads to a significant increase in the magnitude of the association energy as calculated by eq 3. This is somewhat reminiscent of the case of H_2_SO_4_·DMA, which is known to very strongly associate due to the proton transfer taking place already at zero field.^28−30^

**Figure 5:**
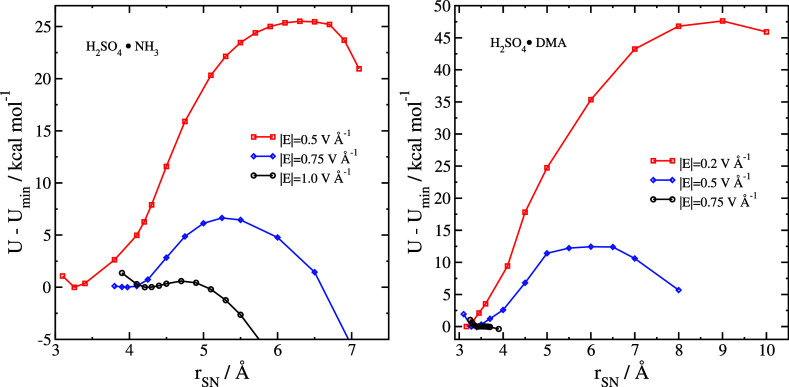
Internal energy *U* – *U*_min_ of HSO_4_^–^·NH_4_^+^ (left) and HSO_4_^–^·DMA^+^ (right) from optimizations constrained to maintain the given separation *r*_SN_ between the nitrogen and sulfur atoms.

As we have already discussed in Section 2.3, however, ion pairs can also dissociate in a high electric field by a process which is not correctly described by the use of eq 3. In Figure 5, we show the result of optimizations constrained to maintain a given separation *r*_SN_ between the sulfur atom in sulfuric acid and the nitrogen atom in ammonia. As the separation is increased from the equilibrium geometry, the energy increases, going through a maximum at the point where the electric field causes the ions to dissociate. We can interpret the height of this barrier as representing an alternate association energy.

**Figure 6:**
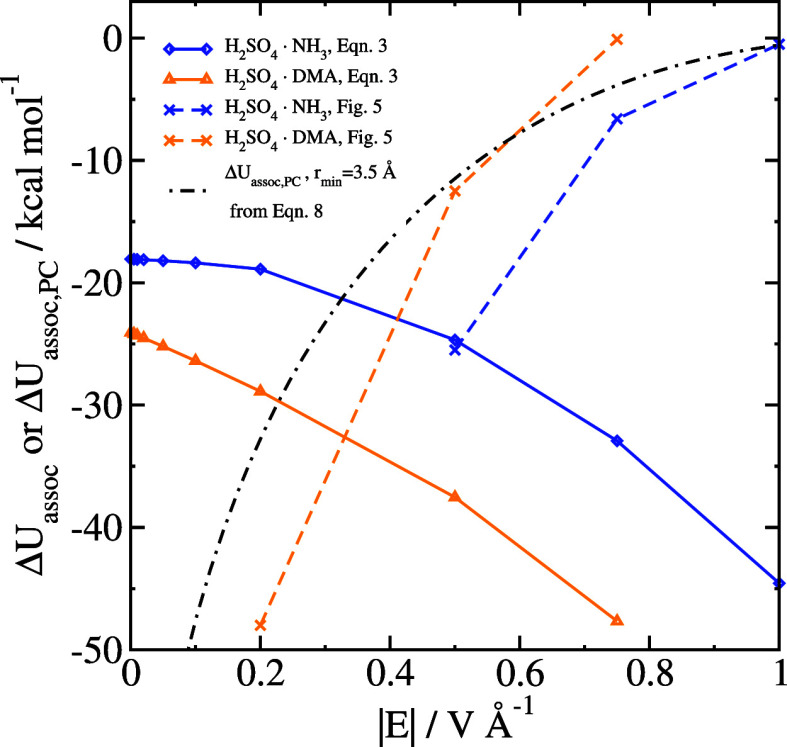
Association energy for systems which form ion pairs, including the standard calculation Δ*U*_assoc_ given in eq 3, results from Figure 5, and comparison to eq 8.

In Figure 6, we compare the results of eq 3 with the barrier heights shown in Figure 5. For |*E*| = 1.0 V Å^–1^, the ion pair is bound by less than 1 kcal mol^–1^. At |*E*| = 0.5 V Å^–1^, the ionic dissociation barrier is quite similar to the value of Δ*U*_assoc_ coming from eq 3. The association energy in different field strengths should be the one of these values that is smaller in magnitude. For the H_2_SO_4_·NH_3_ case, this occurs close to |*E*| = 0.5 V Å^–1^, which is coincidentally the smallest field strength we studied where the ion pair is stabilized.

We also compare our explicit calculation of the ionic dissociation barrier with the point-charge model we described in Section 2.3 and especially eq 8. This is a very crude model; molecular ions are not point charges, and *r*_min_ is only roughly comparable to the equilibrium value of *r*_SN_. Nevertheless, the model manages to qualitatively describe the observed trend.

### 3.4 H_2_SO_4_·DMA Cluster

Our estimate of *U*_assoc_ is −24.1 kcal/mol with the cc-pVTZ basis set in zero electric field. This agrees well with an estimate of −23.9 kcal/mol using either cc-pVTZ or aug-cc-pVTZ basis sets and the same density functional.^29^ As briefly mentioned already in the section on H_2_SO_4_·NH_3_, the H_2_SO_4_·DMA cluster is a special case in that even in zero field a proton transfer reaction is energetically favored, so that the global minimum configuration is an ion pair.^28−30^ Furthermore, the alignment of the molecules produces a large dipole moment [|μ| = 8.504 D]. As a result, out of all the systems we studied, this one shows the largest increase in association energy as a function of field strength.

For the largest field strength we used, |*E*| = 1.0 V Å^–1^, and the field strength is high enough to dissociate the ion pair in an unconstrained simulation. At |*E*| = 0.75 V Å^–1^, the system is barely bound. As already described for H_2_SO_4_·NH_3_, at lower fields, there is a higher barrier for the field-driven ionic dissociation. Since the equilibrium separation between the ions in H_2_SO_4_·DMA is smaller than that in H_2_SO_4_·NH_3_, overall the ionic dissociation barrier is smaller in magnitude for a given field strength. The value of |*E*| at which the ionic dissociation would start to be favored over the neutral dissociation is between 0.2 and 0.5 V Å^–1^, with the most negative value of Δ*U*_assoc_ being approximately −30 to −35 kcal mol^–1^.

### 3.5 H_2_SO_4_ Dimer

**Figure 7:**
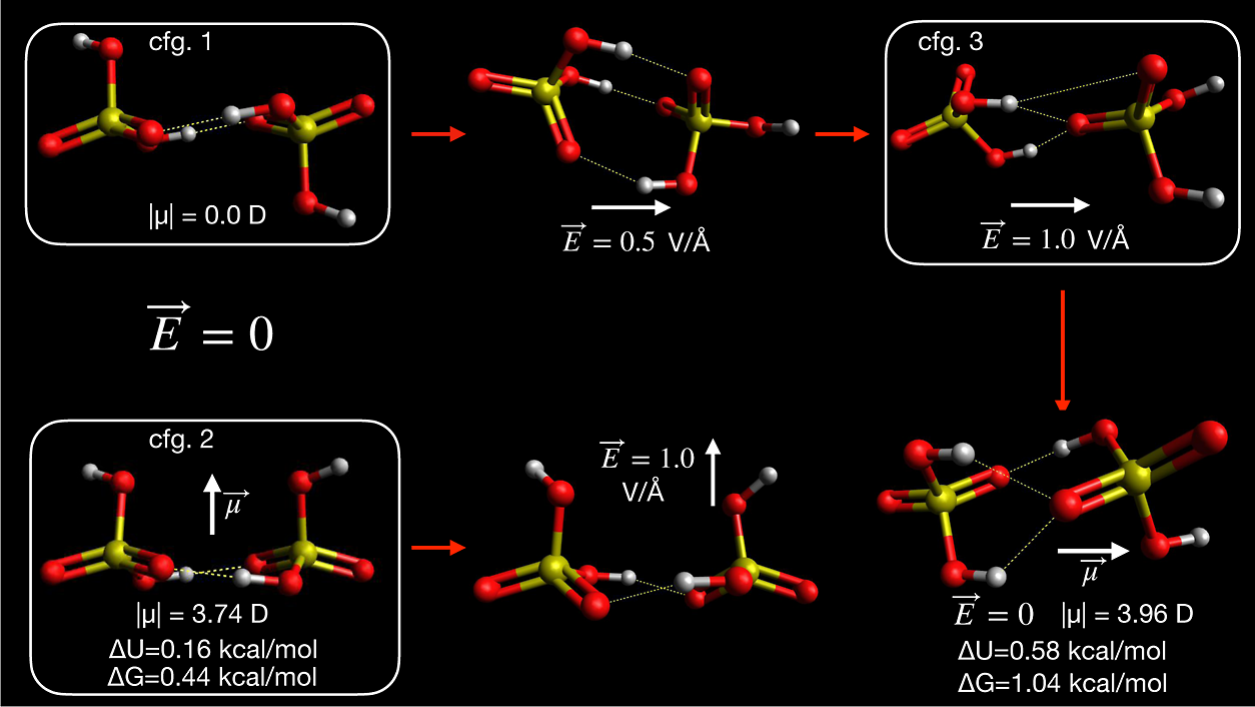
Representative configurations of sulfuric acid dimers. Energies given are relative to the global minimum energy structure (configuration 1). Red arrows indicate the path taken by the optimizations from the initial to final configuration. White arrows indicate the direction of the dipole moment or the electric field vector.

The minimum energy configuration of the sulfuric acid dimer is a cyclic structure with two strong hydrogen bonds. However, there are two possible configurations, one with each dangling OH group on different sides (configuration 1) and one with them on the same side (configuration 2). These are shown in Figure 7. Both of these structures have previously been reported as the global minimum.^30,31^ They are clearly very close in energy, and which one comes out as the global minimum depends on the details of the calculation. In this work, we agree with ref (30) in finding that the configuration with the two dangling OH’s on opposite sides is the global minimum but only by ∼0.2 kcal mol^–^^1^.

**Figure 8:**
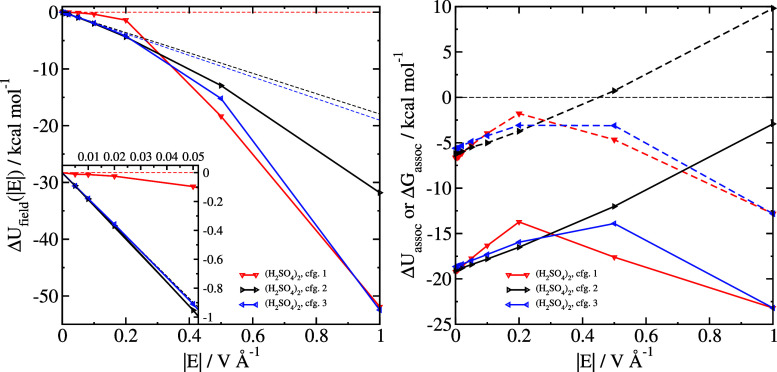
Field response Δ*U*_field_ (left) and field-dependent association energies Δ*U*_assoc_ (open symbols and solid lines) and Δ*G*_assoc_ (closed symbols and dashed lines) (right). Each figure is derived from optimizations starting from three different initial configurations of (H_2_SO_4_)_2_, configuration 1 (global minimum), configuration 2 (alternate cyclic structure), and configuration 3 (optimization in strong field), all shown in Figure 7.

Since these two configurations are so close in energy, it is likely that both coexist among atmospheric populations of sulfuric acid dimers. However, these configurations have much different dipole moments, so their responses to the electric field should differ also. In Figure 8, it can be seen that the field response of configuration 1 in low field is much smaller than that of configuration 2. Since configuration 1 has zero dipole moment in zero field owing to symmetry, the only field response possible is an induced dipole. However, in higher field (|*E*| ≥ 0.5 V Å^–1^), we see drastic changes to the geometry of configuration 1, allowing the system to reduce its overall energy compared with configuration 2 at the same field strength. Overall, we can conclude that structures resembling configuration 2 are the global minimum for this system in field strengths between 0.05 and 0.2 V Å^–1^. The raw data used to make Figure 8 are also tabulated in Tables 1 and 2.

**Table 2:** Table of Data for Sulfuric Acid Dimers, with the Optimizations Starting from Different Initial Conformations Shown in Figure 7^a^

|*E*|/V Å^–1^	μ/D	*U*_field_ – *U*_0_/kcal mol^–1^	*S* × 298.15 K/kcal mol^–1^	*G*_field_ – *G*_0_/kcal mol^–1^	Δ*U*_assoc_/kcal mol^–1^	Δ*G*_assoc_/kcal mol^–1^
(H_2_SO_4_)_2_, cfg. 2						
0	3.736	0	30.57	0	–19.043	–6.212
0.005		–0.090	30.41	0.082	–18.980	–6.109
0.01		–0.182	30.48	–0.078	–18.919	–6.096
0.02		–0.369	30.45	–0.232	–18.797	–5.951
0.05		–0.953	30.38	–0.764	–18.427	–5.483
0.1		–2.008	30.39	–1.864	–17.809	–5.015
0.2		–4.355	30.23	–4.066	–16.510	–3.731
0.5		–12.919	30.02	–12.487	–12.015	0.723
1.0		–31.806	29.83	–31.325	–2.926	9.808
(H_2_SO_4_)_2_, cfg. 3						
0	3.963	0	30.43	0	–18.640	–5.611
0.005		–0.092	30.37	–0.009	–18.579	–5.598
0.01		–0.180	30.36	–0.094	–18.513	–5.510
0.02		–0.356	30.34	–0.227	–18.382	–5.344
0.05		–0.910	30.32	–0.739	–17.981	–4.856
0.1		–1.906	30.28	–1.683	–17.304	–4.233
0.2		–4.202	30.30	–4.022	–15.954	–3.085
0.5		–15.209	32.11	–16.930	–13.901	–3.118
1.0		–52.484	31.86	–54.555	–23.200	–12.820

a All headings and details are the same as those in Table 1.

(H_2_SO_4_)_2_ has other local minima in addition to the cyclic structures. For example, if configuration 1 is reoptimized in a field of 1.0 V Å^–1^ and that configuration is then used as the initial structure and reoptimized in zero field, another structure shown in Figure 7 is obtained, which is higher in energy than the global minimum by only ∼0.6 kcal mol^–1^. Therefore, we also decided to investigate what happens if this asymmetric structure (configuration 3) is used as the starting point for optimizations in different field strengths. We can see that such a structure finds another local minimum at |*E*| = 0.5 V Å^–1^, which resembles the configuration reached by configuration 2 at the same field strength. At lower field strength, we can see that the energies are quite close to those obtained starting from configuration 2. This is sensible since the dipole moment magnitudes of configuration 2 and configuration 3 optimized in zero field are quite similar, although the geometries and the direction of the dipole moment are quite different.

## 4 Conclusions

We have used DFT-based quantum chemical methods and geometry optimizations to study the impact of external electric fields on the structure and energetics of important bimolecular atmospheric clusters, including (H_2_O)_2_ as well as clusters of sulfuric acid with H_2_O, NH_3_, DMA, and sulfuric acid dimers.

Based on our results, it is clear that for electric field strengths up to 0.02 V Å^–1^ or so, the effect of an electric field on a cluster may be predicted well by simply computing the dipole moments of each molecule separately and the dipole moment of the cluster, in zero field, followed by application of eq 10. Depending on the geometry of the cluster, this can cause the association energies Δ*U*_assoc_ and Δ*G*_assoc_ to increase or decrease compared to the field-free association energies.

Strongly bound acid–base clusters such as H_2_SO_4_·DMA which form ion pairs and have large dipole moments will therefore be more strongly bound in small electric fields, while in other cases, such as clusters including organic molecules, association energies could either increase or decrease. However, the magnitude of these changes is minimal (<0.5 kcal mol^–1^) for the smallest field strengths we studied, which are already orders of magnitude larger than naturally occurring electric fields in the atmosphere, or the ion-clearing fields used in the CERN CLOUD chamber. For example, according to eq 10, we would only expect the association energy of H_2_SO_4_·DMA to increase in magnitude by 6.3 × 10^–5^ kcal mol^–1^ due to a clearing field of 0.3 × 10^–5^ V Å^–1^.

In clusters of more complex molecules such as the sulfuric acid dimer, there are many local minima which are close in energy but have very different dipole moments. Therefore, as the electric field strength is increased, the differing field response of each conformation can change which minimum is the global minimum. The number of conformers with similar energies would increase more in larger clusters. Again, we stress that in almost all experimental or natural situations, the effect of the field would be negligible. However, consider that there could be multiple decomposition channels available for a cluster consisting of a few molecules. Near an extended interface, such as a larger aerosol or an ice particle, the substantial field generated by the interface could promote decomposition pathways in which the total dipole moment of the system increases and vice versa. Such effects are worth looking out for as aerosol science progresses to study more complex systems.

In extremely large fields, such as those seen near a solid or liquid interface or caused by a laser, in excess of 0.2 V Å^–1^, new effects become important. Geometric distortion and induced dipoles tend to increase the magnitude of the association energy due to strong head–tail dipole interactions in the clusters. In some cases, such as H_2_SO_4_·NH_3_, the high field can induce a proton transfer reaction. Ion pairing in this case, as well as in H_2_SO_4_·DMA, in combination with the field adds new physics that must be considered. An ionic dissociation driven by the field becomes relevant, and depending on the system this barrier can be lower than the association energy calculated for two neutral molecules for |*E*| > 0.2 V Å^–1^.

## Supporting Information Available

Results of test calculations comparing two methods of applying an electric field in quantum chemical calculations; impact of including polarization in the model for dipolar response to the electric field; energies as a function of field strength for single molecules; results obtained with the aug-cc-pVTZ basis set to compare with the cc-pVTZ basis set, including some example timings for doing numerical frequency calculations; and results obtained with the D2 dispersion correction to compare with the D3 correction (PDF)
ORCA input files and optimized configurations for a few representative clusters (ZIP)

## References

[ref1] Trinh T. N.; Scholten O.; Buitink S.; Ebert U.; Hare B. M.; Krehbiel P. R.; Leijnse H.; Bonardi A.; Corstanje A.; Falcke H.; et al. Determining Electric Fields in Thunderclouds With the Radiotelescope LOFAR. J. Geophys. Res.: Atmos. 2020, 125, e2019JD031433 10.1029/2019jd031433. PMC7375151 32714723

[ref2] Tröstl J.; Chuang W. K.; Gordon H.; Heinritzi M.; Yan C.; Molteni U.; Ahlm L.; Frege C.; Bianchi F.; Wagner R.; et al. The role of low-volatility organic compounds in initial particle growth in the atmosphere. Nature 2016, 533, 527–531. 10.1038/nature18271. 27225126 PMC8384036

[ref3] Pedersen J. O. P.; Enghoff M. B.; Paling S. M.; Svensmark H. Aerosol nucleation in an ultra-low ion density environment. J. Aerosol Sci. 2012, 50, 75–85. 10.1016/j.jaerosci.2012.03.003.

[ref4] Maher S.; Jjunju F. P. M.; Taylor S. Colloquium: 100 years of mass spectrometry: Perspectives and future trends. Rev. Mod. Phys. 2015, 87, 113–135. 10.1103/RevModPhys.87.113.

[ref5] Fenn J. B. Electrospray Wings for Molecular Elephants (Nobel Lecture). Angew. Chem., Int. Ed. 2003, 42, 3871–3894. 10.1002/anie.200300605. 12949861

[ref6] Li H.; Almeida T. G.; Luo Y.; Zhao J.; Palm B. B.; Daub C. D.; Huang W.; Mohr C.; Krechmer J. E.; Kurtén T.; et al. Fragmentation inside proton-transfer-reaction-based mass spectrometers limits the detection of ROOR and ROOH peroxides. Atmos. Meas. Technol. 2022, 15, 1811–1827. 10.5194/amt-15-1811-2022.

[ref7] Thomson B. A.; Iribarne J. V. Field induced ion evaporation from liquid surfaces at atmospheric pressure. J. Chem. Phys. 1979, 71, 4451–4463. 10.1063/1.438198.

[ref8] Fenn J. B.; Rosell J.; Meng C. K. In Electrospray Ionization, How Much Pull Does an Ion Need to Escape Its Droplet Prison?. J. Am. Soc. Mass Spectrom. 1997, 8, 1147–1157. 10.1016/S1044-0305(97)00161-X.

[ref9] Bresme F.; Chacón E.; Tarazona P.; Wynveen A. The structure of ionic aqueous solutions at interfaces: An intrinsic structure analysis. J. Chem. Phys. 2012, 137, 114706 10.1063/1.4753986. 22998280

[ref10] Angle K. J.; Nowak C. M.; Grassian V. H. Organic acid evaporation kinetics from aqueous aerosols: implications for aerosol buffering capacity in the atmosphere. Environ. Sci.: Atmos. 2023, 3, 316–327. 10.1039/d2ea00092j.

[ref11] Daub C. D.; Bratko D.; Luzar A. Nanoscale Wetting Under Electric Field from Molecular Simulations. Top. Curr. Chem. 2011, 307, 155–179. 10.1007/128_2011_188. 21769717

[ref12] English N. J.; Waldron C. J. Perspectives on external electric fields in molecular simulation: progress, prospects and challenges. Phys. Chem. Chem. Phys. 2015, 17, 12407–12440. 10.1039/c5cp00629e. 25903011

[ref13] Vegiri A. Reorientational relaxation and rotational-translational coupling in water clusters in a d.c. external electric field. J. Mol. Liq. 2004, 110, 155–168. 10.1016/j.molliq.2003.09.011.

[ref14] Choi Y. C.; Pak C.; Kim K. S. Electric field effects on water clusters (*n* = 3 – 5): Systematic ab initio study of structures, energetics, and transition states. J. Chem. Phys. 2006, 124, 094308 10.1063/1.2173259. 16526858

[ref15] Mondal A.; Seenivasan H.; Saurav S.; Tiwari A. K. Behavior of water dimer under the influence of external electric fields. Indian J. Chem., Sect. A: Inorg., Bio-inorg., Phys., Theor. Anal. Chem. 2013, 52, 1056–1060.

[ref16] Toledo E. J. L.; Custodio R.; Ramalho T. C.; Porto M. E. G.; Magriotis Z. M. Electrical field effects on dipole moment, structure and energetic of (H_2_O)_n_ (2 ≤ *n* ≤ 15) cluster. J. Mol. Struct.: THEOCHEM 2009, 915, 170–177. 10.1016/j.theochem.2009.08.035.

[ref17] Dubov D. Y.; Vostrikov A. A. Dipole Moment of a Small Water Cluster. The Effect of Size, Temperature, and Electric Field. J. Exp. Theor. Phys. Lett. 2010, 92, 28–32. 10.1134/S0021364010130059.

[ref18] Acosta-Gutiérrez S.; Hernández-Rojas J.; Bretón J.; Llorente J. M. G.; Wales D. J. Physical properties of small water clusters in low and moderate electric fields. J. Chem. Phys. 2011, 135, 124303 10.1063/1.3640804. 21974518

[ref19] Chakraborty S. N.; English N. J. Vibrational, energetic-dynamical and dissociation properties of water clusters in static electric fields: Non-equilibrium molecular-dynamics insights. Chem. Phys. Lett. 2018, 710, 207–214. 10.1016/j.cplett.2018.08.061.

[ref20] Vu N. H.; Dong H. C.; Nguyen M. V.; Hoang D.; Trinh T. T.; Phan T. B. Mechanism of proton transport in water clusters and the effect of electric fields: A DFT study. Curr. Appl. Phys. 2021, 25, 62–69. 10.1016/j.cap.2021.02.006.

[ref21] Rai D.; Kulkarni A. D.; Gejji S. P.; Pathak R. K. Methanol clusters (CH_3_OH)_n_, *n* = 3 – 6 in external electric fields: Density functional theory approach. J. Chem. Phys. 2011, 135, 024307 10.1063/1.3605630. 21766942

[ref22] Sarmah N.; Bhattacharyya P. K. Behaviour of cation–pi interaction in presence of external electric field. RSC Adv. 2016, 6, 100008–100015. 10.1039/C6RA21334K.

[ref23] Meir R.; Chen H.; Lai W.; Shaik S. Oriented Electric Fields Accelerate Diels–Alder Reactions and Control the endo/exo Selectivity. ChemPhysChem 2010, 11, 301–310. 10.1002/cphc.200900848. 19998402

[ref24] Daub C. D.; Cann N. M. How Are Completely Desolvated Ions Produced in Electrospray Ionization: Insights from Molecular Dynamics Simulations. Anal. Chem. 2011, 83, 8372–8376. 10.1021/ac202103p. 22017403

[ref25] Daub C. D.; Åstrand P.-O.; Bresme F. Lithium Ion-Water Clusters in Strong Electric Fields: A Quantum Chemical Study. J. Phys. Chem. A 2015, 119, 4983–4992. 10.1021/acs.jpca.5b01822. 25918829

[ref26] Cramer T.; Zerbetto F.; García R. Molecular Mechanism of Water Bridge Buildup: Field-Induced Formation of Nanoscale Menisci. Langmuir 2008, 24, 6116–6120. 10.1021/la800220r. 18484756

[ref27] Kurtén T.; Noppel M.; Vehkamäki H.; Salonen M.; Kulmala M. Quantum chemical studies of hydrate formation of H_2_SO_4_ and HSO_4_^–^. Boreal Environ. Res. 2007, 12, 431–453.

[ref28] Myllys N.; Elm J.; Halonen R.; Kurtén T.; Vehkamäki H. Coupled Cluster Evaluation of the Stability of Atmospheric Acid-Base Clusters with up to 10 Molecules. J. Phys. Chem. A 2016, 120, 621–630. 10.1021/acs.jpca.5b09762. 26771121

[ref29] Myllys N.; Elm J.; Kurtén T. Density functional theory basis set convergence of sulfuric acid-containing molecular clusters. Comput. Theor. Chem. 2016, 1098, 1–12. 10.1016/j.comptc.2016.10.015.

[ref30] Myllys N.; Kubečka J.; Besel V.; Alfaouri D.; Olenius T.; Smith J. N.; Passananti M. Role of base strength, cluster structure and charge in sulfuric-acid-driven particle formation. Atmos. Chem. Phys. 2019, 19, 9753–9768. 10.5194/acp-19-9753-2019.

[ref31] Elm J.; Kristensen K. Basis set convergence of the binding energies of strongly hydrogen-bonded atmospheric clusters. Phys. Chem. Chem. Phys. 2017, 19, 1122–1133. 10.1039/C6CP06851K. 27942640

[ref32] Elm J.; Kubečka J.; Besel V.; Jääskeläinen M. J.; Halonen R.; Kurtén T.; Vehkamäki H. Modeling the formation and growth of atmospheric molecular clusters: A review. J. Aerosol Sci. 2020, 149, 105621 10.1016/j.jaerosci.2020.105621.

[ref33] Engsvang M.; Wu H.; Knattrup Y.; Kubečka J.; Jensen A. B.; Elm J. Quantum chemical modeling of atmospheric molecular clusters involving inorganic acids and methanesulfonic acid. Chem. Phys. Rev. 2023, 4, 031311 10.1063/5.0152517.

[ref34] Neese F. The ORCA program system. Wiley Interdiscip. Rev.: Comput. Mol. Sci. 2012, 2, 73–78. 10.1002/wcms.81.

[ref35] Neese F. Software update: the ORCA program system, version 4.0. Wiley Interdiscip. Rev.: Comput. Mol. Sci. 2017, 8, e1327 10.1002/wcms.1327.

[ref36] Chai J.-D.; Head-Gordon M. Systematic optimization of long-range corrected hybrid density functionals. J. Chem. Phys. 2008, 128, 084106 10.1063/1.2834918. 18315032

[ref37] Chai J.-D.; Head-Gordon M. Long-range corrected hybrid density functionals with damped atom–atom dispersion corrections. Phys. Chem. Chem. Phys. 2008, 10, 6615–6620. 10.1039/b810189b. 18989472

[ref38] Lin Y.-S.; Li G.-D.; Mao S.-P.; Chai J.-D. Long-Range Corrected Hybrid Density Functionals with Improved Dispersion Corrections. J. Chem. Theory Comput. 2013, 9, 263–272. 10.1021/ct300715s. 26589028

[ref39] Woon D. E.; Dunning T. H. Gaussian basis sets for use in correlated molecular calculations. IV. Calculation of static electrical response properties. J. Chem. Phys. 1994, 100, 2975–2988. 10.1063/1.466439.

[ref40] Grimme S.; Antony J.; Ehrlich S.; Krieg H. A consistent and accurate *ab initio* parametrization of density functional dispersion correction (DFT-D) for the 94 elements H-Pu. J. Chem. Phys. 2010, 132, 154104 10.1063/1.3382344. 20423165

[ref41] Grimme S.; Ehrlich S.; Goerigk L. Effect of the Damping Function in Dispersion Corrected Density Functional Theory. J. Comput. Chem. 2011, 32, 1456–1465. 10.1002/jcc.21759. 21370243

[ref42] Grimme S. Accurate description of van der Waals complexes by density functional theory including empirical corrections. J. Comput. Chem. 2004, 25, 1463–1473. 10.1002/jcc.20078. 15224390

[ref43] Halkier A.; Koch H.; Jørgensen P.; Christiansen O.; Nielsen I. M. B.; Helgaker T. A systematic ab initio study of the water dimer in hierarchies of basis sets and correlation models. Theor. Chem. Acc. 1997, 97, 150–157. 10.1007/s002140050248.

[ref44] Klopper W.; M van Duijneveldt-van de Rijdt J. G. C.; van Duijneveldt F. B. Computational determination of equilibrium geometry and dissociation energy of the water dimer. Phys. Chem. Chem. Phys. 2000, 2, 2227–2234. 10.1039/a910312k.

[ref45] Ruscic B. Active Thermochemical Tables: Water and Water Dimer. J. Phys. Chem. A 2013, 117, 11940–11953. 10.1021/jp403197t. 23834334

[ref46] Mukhopadhyay A.; Cole W. T. S.; Saykally R. J. The water dimer I: Experimental characterization. Chem. Phys. Lett. 2015, 633, 13–26. 10.1016/j.cplett.2015.04.016.

[ref47] Mukhopadhyay A.; Xantheas S. S.; Saykally R. J. The water dimer II: Theoretical investigations. Chem. Phys. Lett. 2018, 700, 163–175. 10.1016/j.cplett.2018.03.057.

[ref48] Partanen L.; Hänninen V.; Halonen L. Effects of Global and Local Anharmonicities on the Thermodynamic Properties of Sulfuric Acid Monohydrate. J. Chem. Theory Comput. 2016, 12, 5511–5524. 10.1021/acs.jctc.6b00683. 27662456

[ref49] Schmitz G.; Elm J. Assessment of the DLPNO Binding Energies of Strongly Noncovalent Bonded Atmospheric Molecular Clusters. ACS Omega 2020, 5, 7601–7612. 10.1021/acsomega.0c00436. 32280904 PMC7144154

[ref50] Hanson D. R.; Eisele F. Diffusion of H_2_SO_4_ in Humidified Nitrogen: Hydrated H_2_SO_4_. J. Phys. Chem. A 2000, 104, 1715–1719. 10.1021/jp993622j.

