# Supplementary material for: Effect of an Electric Field on the Structure and Stability of Atmospheric Clusters
Source: J Phys Chem A. 2024 Jan 13;128(3):646–55. doi: 10.1021/acs.jpca.3c07260 (PMC11389980; doi:10.1021/acs.jpca.3c07260)
Supplement: Supplementary file 1 — jp3c07260_si_001.pdf [file jp3c07260_si_001.pdf]

Supporting Information: The Effect of an Electric Field on the Structure and Stability of Atmospheric Clusters

Christopher David Daub* and Theo Kurtén*

Department of Chemistry, University of Helsinki, P.O. Box 55, Helsinki 00014, Finland

E-mail: christopher.daub@helsinki.fi; theo.kurten@helsinki.fi

Comparison of Two Methods for Applying an Electric Field

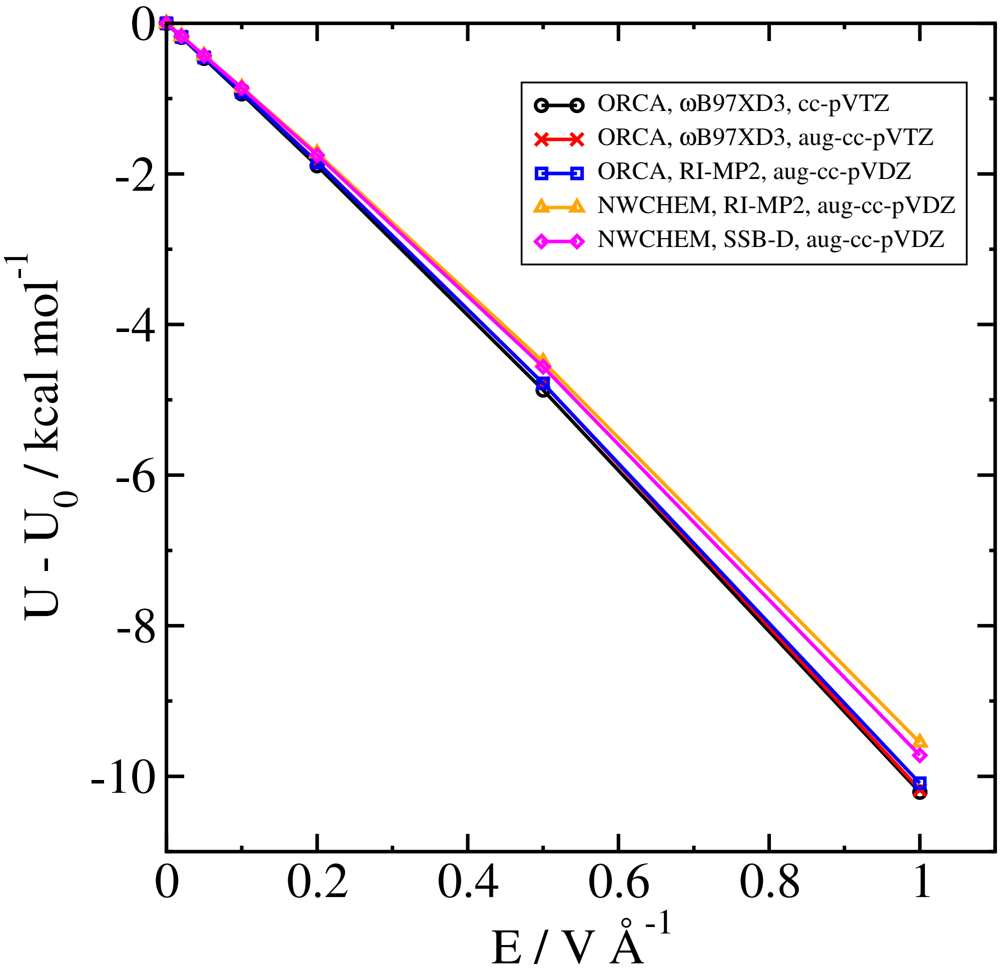

Figure S1: Comparison of the field response of one H_2O molecule, optimized using either ORCA[S1] with electric field applied directly, or NWCHEM[S2] with electric field approximated by point charges placed ±30 Å from the center of the oxygen atom. The NWCHEM optimizations were constrained to fix the position of the oxygen atom.

Effect of Including a Polarization Term

In Equation 9 of the main text, we model the field response of our systems simply as a linear response to the permanent, zero-field dipole moment μ. We can include an induced dipole by adding the polarizability α and modelling the field response as

$$U_{\text{field}} - U_0 = -\mu_{tot}E = -(\mu + \alpha E)E = -\mu E - \alpha E^2. \tag{1}$$

The polarizability α is a tensor, but for our purposes we will simply assume it is isotropic so that we can express α as a single scalar quantity.

We computed polarizabilities with ORCA and in Figure S2 we show a version of Figure 1 from the main text, but including new results using the model of Equation S1 above. Quantitatively, inclusion of polarizability does improve agreement between our direct measurements and the dipole model. However, it is not very systematic. Inclusion of polarizability leads to an overestimate of the field response in most of the single molecules. In the bimolecular clusters, Equation S1 still under-estimates the field response, which we can attribute to the significant impact of field-driven molecular deformation on the cluster geometries, which is not included in models based on zero-field measurements.

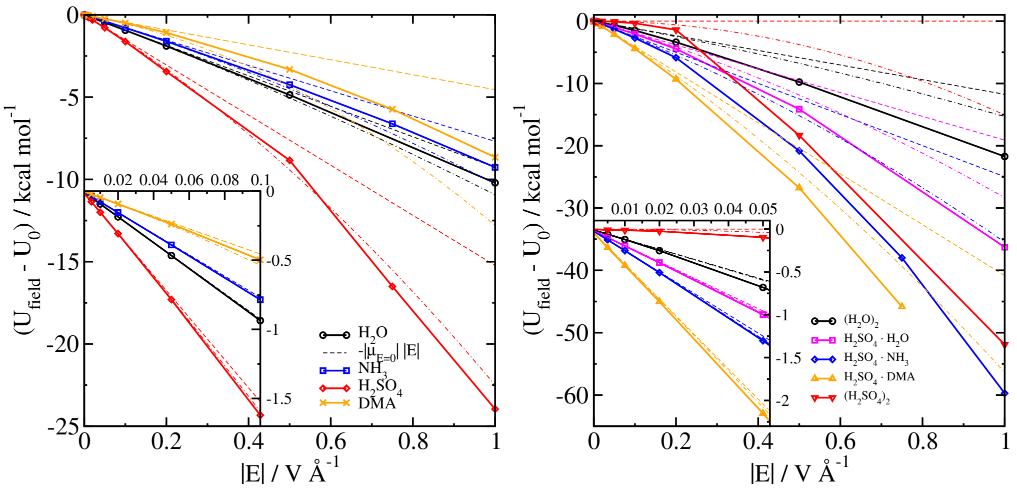

Figure S2: Figure 1 in the main text, but with added results using Equation S1 including the isotropic polarizability (dash-dotted lines).

A similar exercise starting from Equation 10 in the main text gives us a way to include polarizability in an expression for the field dependence of the binding energy:

$$\Delta U_{\text{assoc}} - \Delta U_{\text{assoc},0} = -\Delta\mu|E| - \Delta\alpha|E|^2. \tag{2}$$

Here $\Delta\alpha = \alpha_{\text{clust}} - \sum_1^n \alpha_i$ is defined the same way as $\Delta\mu$ in the main text. Results from using Equation S2 instead of Equation 10 are shown in Figure S3. In all of the cases we studied, it turns out that $\Delta\alpha$ is quite small, owing to near-cancellation of the polarizabilites of the cluster and the constituent molecules, so that there is almost no effect from including the polarizability in Equation S2.

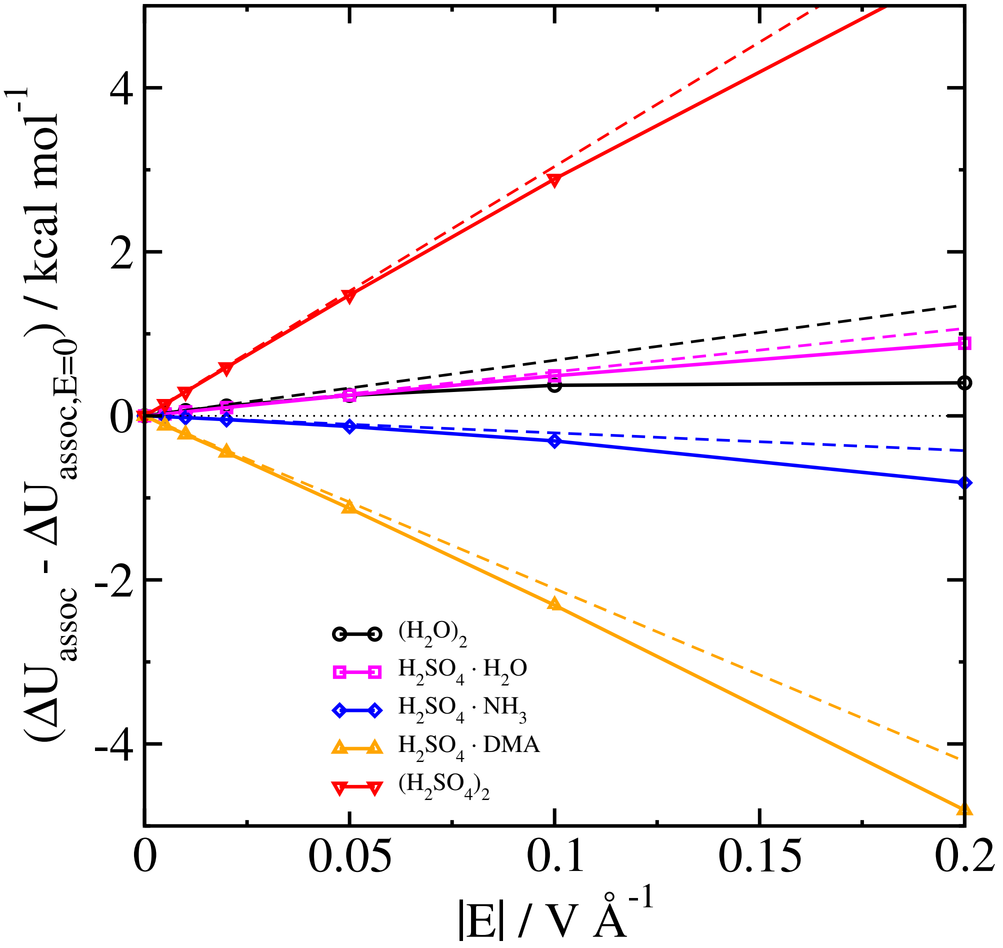

Figure S3: Similar to Figure 3 in the main text, but using Equation S2 including the change in isotropic polarizability $\Delta\alpha$ (dashed lines) to model the change in binding energy with field strength.

Table of Optimized Energies for Single Molecules

Table S1: Electronic energies $U_{\text{field}} - U_0$, dipole moment μ, entropy term TS, and Gibbs free energies $G_{\text{field}} - G_0$ for single molecules as a function of the electric field strength $|E|$, referred to results in zero field. All optimizations were done with the ωB97X functional[S3–S5] including Grimme's D3 dispersion correction,[S6,S7] and the cc-pVTZ basis set.

$|E|$/V Å^{-1}	μ / D	$U_{\text{field}} - U_0$ /kcal mol^{-1}	$S \times 298.15$ K /kcal mol^{-1}	$G_{\text{field}} - G_0$ /kcal mol^{-1}
H_2O				
0	1.929	0	13.43	0
0.005		-0.046	13.43	-0.046
0.01		-0.093	13.43	-0.093
0.02		-0.186	13.43	-0.186
0.05		-0.466	13.44	-0.467
0.1		-0.936	13.44	-0.938
0.2		-1.891	13.44	-1.896
0.5		-4.870	13.44	-4.886
1.0		-10.211	13.44	-10.262
NH_3				
0	1.601	0	13.70	0
0.005		-0.039	13.70	-0.038
0.01		-0.077	13.70	-0.075
0.02		-0.155	13.70	-0.149
0.05		-0.389	13.70	-0.377
0.1		-0.786	13.70	-0.763
0.2		-1.603	13.70	-1.562
0.5		-4.245	13.70	-4.157
0.75		-6.620	13.70	-6.513
1.0		-9.264	13.69	-9.126
DMA ($(CH_3)_2NH$)				
0	0.947	0	19.36	0
0.005		-0.023	19.36	-0.021
0.01		-0.046	19.36	-0.041
0.02		-0.093	19.36	-0.085
0.05		-0.238	19.36	-0.218
0.1		-0.497	19.36	-0.466
0.2		-1.077	19.36	-1.023
0.5		-3.313	19.37	-3.215
0.75		-5.735	19.38	-5.633
1.0		-8.658	19.39	-8.581
H_2SO_4				
0	3.172	0	21.14	0
0.005		-0.077	21.09	-0.011
0.01		-0.153	21.10	-0.097
0.02		-0.307	21.10	-0.247
0.05		-0.784	21.11	-0.747
0.1		-1.621	21.06	-1.531
0.2		-3.444	21.00	-3.274
0.5		-8.844	20.89	-9.711
0.75		-16.508	20.81	-16.180
1.0		-23.96	20.78	-23.67

Results With The aug-cc-pVTZ Basis Set

Table S2: Electronic energies $U_{\mathrm{field}} - U_0$, dipole moment μ, entropy term TS, and Gibbs free energies $G_{\mathrm{field}} - G_0$ for single molecules as a function of the electric field strength $|E|$. All calculation details are identical with Table S1 except for using the aug-cc-pVTZ basis set.

$\lvert E\rvert$/V Å^{-1}	μ / D	$U_{\mathrm{field}} - U_0$ /kcal mol^{-1}	$S \times 298.15$ K /kcal mol^{-1}	$G_{\mathrm{field}} - G_0$ /kcal mol^{-1}
H_2O				
0	1.863	0	13.43	0
0.005		-0.045	13.43	-0.045
0.01		-0.090	13.43	-0.090
0.02		-0.180	13.43	-0.180
0.05		-0.451	13.43	-0.451
0.1		-0.907	13.44	-0.909
0.2		-1.839	13.44	-1.844
0.5		-4.780	13.44	-4.800
1.0		-10.176	13.45	-10.237
NH_3				
0	1.524	0	13.70	0
0.005		-0.036	13.70	-0.036
0.01		-0.073	13.70	-0.071
0.02		-0.147	13.70	-0.142
0.05		-0.372	13.70	-0.359
0.1		-0.754	13.70	-0.730
0.2		-1.554	13.70	-1.508
0.5		-4.216	13.70	-4.117
1.0		-9.507	13.70	-9.362
DMA				
0	1.010	0	19.36	0
0.005		-0.025	19.36	-0.022
0.01		-0.049	19.36	-0.045
0.02		-0.099	19.36	-0.092
0.05		-0.254	19.36	-0.235
0.1		-0.531	19.36	-0.495
0.2		-1.155	19.36	-1.089
0.5		-3.573	19.36	-3.451
1.0		-9.422	19.37	-9.303
H_2SO_4				
0	3.138	0	21.16	0
0.005		-0.076	21.12	-0.023
0.01		-0.151	21.10	-0.073
0.02		-0.305	21.13	-0.273
0.05		-0.778	21.12	-0.729
0.1		-1.611	21.09	-1.535
0.2		-3.436	21.02	-3.274
0.5		-10.046	20.93	-9.812
1.0		-24.432	20.81	-24.181

Table S3: Electronic energies $U_{\text{field}} - U_0$, dipole moment μ, entropy term TS, Gibbs free energies $G_{\text{field}} - G_0$, and electronic association energies ΔU_{assoc} and Gibbs free energy for association ΔG_{assoc} as a function of the electric field strength $|E|$ for clusters. All calculation details are identical with Table S2. Results in parentheses include BSSE.

$\lvert E \rvert$/V Å^{-1}	μ / D	$U_{\text{field}} - U_0$ /kcal mol^{-1}	$S \times 298.15$ K /kcal mol^{-1}	$G_{\text{field}} - G_0$ /kcal mol^{-1}	ΔU_{assoc} /kcal mol^{-1}	ΔG_{assoc} /kcal mol^{-1}
$(H_2O)_2$						
0	2.534	0	20.77	0	-5.036(-4.982)	2.686
0.005		-0.062	20.77	-0.060	-5.007	2.716
0.01		-0.125	20.75	-0.105	-4.981	2.760
0.02		-0.256	20.72	-0.203	-4.933	2.842
0.05		-0.683	20.69	-0.605	-4.818	2.984
0.1		-1.487	20.67	-1.394	-4.708	3.111
0.2		-3.326	20.64	-3.194	-4.684	3.180
0.5		-9.612	20.16	-8.939	-5.087	3.348
1.0		-21.598	19.68	-20.393	-6.281(-6.218)	2.767
$H_2SO_4 \cdot H_2O$						
0	3.994	0	25.54	0	-12.609(-12.415)	-1.960
0.005		-0.099	25.50	-0.044	-12.587	-1.937
0.01		-0.196	25.47	-0.1067	-12.563	-1.904
0.02		-0.392	25.45	-0.287	-12.516	-1.795
0.05		-0.998	25.54	-0.855	-12.378	-1.774
0.1		-2.073	25.46	-1.977	-12.162	-1.493
0.2		-4.488	25.56	-4.504	-11.822	-1.347
0.5		-14.425	25.82	-14.838	-12.207	-2.186
1.0		-36.998	25.40	-37.384	-14.998(-14.803)	-4.927
$H_2SO_4 \cdot NH_3$						
0	5.257	0	26.01	0	-16.610	-6.409
0.005		-0.129			-16.626	
0.01		-0.255			-16.640	
0.02		-0.511	25.93	-0.440	-16.669	-6.435
0.05		-1.308			-16.768	
0.1		-2.723			-16.967	
0.2		-5.911			-17.531	
0.5		-21.920			-24.268	
1.0		-62.735			-45.405	
$H_2SO_4 \cdot$DMA						
0	8.621	0	30.00	0	-24.057	-11.015
$(H_2SO_4)_2$						
0	0.0	0	30.84	0	-18.332	-5.710

Table S4: CPU hours for a single numerical frequency calculation for different systems using different basis sets. All calculations used the ωB97X functional with the Grimme D3 dispersion correction, and were run with ORCA on one node of the Mahti supercomputer at Finland's CSC.

System	CPU hours: cc-pVTZ	aug-cc-pVTZ
DMA	165 h	420 h
$H_2SO_4 \cdot H_2O$	320 h	820 h
$H_2SO_4 \cdot NH_3$	380 h	1050 h
$H_2SO_4 \cdot$DMA	1570 h	
$(H_2SO_4)_2$	1250 h	

Results With the D2 Dispersion Correction

Table S5: Results of calculations with the ωB97X functional and the cc-pVTZ basis set, and Grimme's empirical D2 dispersion correction,[S8] for water, sulfuric acid, and the $H_2SO_4{\cdot}H_2O$ cluster.

$\|E\|$/V Å^{-1}	μ / D	$U_{\text{field}} - U_0$ /kcal mol^{-1}	$S \times 298.15$ K /kcal mol^{-1}	$G_{\text{field}} - G_0$ /kcal mol^{-1}	ΔU_{assoc} /kcal mol^{-1}	ΔG_{assoc} /kcal mol^{-1}
H_2O						
0	1.935	0	13.44	0		
0.005		-0.046	13.44	-0.046		
0.01		-0.093	13.44	-0.093		
0.02		-0.186	13.44	-0.186		
0.05		-0.467	13.44	-0.468		
0.1		-0.936	13.44	-0.941		
0.2		-1.896	13.44	-1.901		
0.5		-4.883	13.44	-4.900		
1.0		-10.237	13.45	-10.289		
H_2SO_4						
0	3.210	0	21.12	0		
0.005		-0.077	21.08	-0.029		
0.01		-0.155	21.09	-0.117		
0.02		-0.311	21.09	-0.277		
0.05		-0.793	21.09	-0.762		
0.1		-1.638	21.04	-1.549		
0.2		-3.470	21.01	-3.360		
0.5		-10.006	20.91	-9.834		
1.0		-23.994	20.80	-23.802		
$H_2SO_4{\cdot}H_2O$						
0	4.060	0	25.29	0	-17.866	-6.815
0.005		-0.098	25.27	-0.070	-17.841	-6.782
0.01		-0.196	25.27	-0.167	-17.815	-6.772
0.02		-0.395	25.23	-0.315	-17.764	-6.667
0.05		-1.008	25.19	-0.887	-17.615	-6.433
0.1		-2.087	25.28	-2.107	-17.376	-6.433
0.2		-4.459	25.21	-4.422	-16.959	-5.976
0.5		-13.243	25.12	-13.204	-16.221	-5.286
1.0		-35.319	25.34	-36.035	-18.955	-8.760

References

(S1) Neese, F. Software update: the ORCA program system, version 4.0. *WIREs Comput. Mol. Sci.* **2017**, *8*, e1327.

(S2) Aprà, E.; Bylaska, E. J.; de Jong, W. A.; Govind, N.; Kowalski, K.; Straatsma, T. P.;

Valiev, M.; van Dam, H. J. J.; Alexeev, Y.; Anchell, J.; Anisimov, V.; Aquino, F. W.; Atta-Fynn, R.; Autschbach, J.; Bauman, N. P.; Becca, J. C.; Bernholdt, D. E.; Bhaskaran-Nair, K.; Bogatko, S.; Borowski, P.; Boschen, J.; Brabec, J.; Bruner, A.; Cauët, E.; Chen, Y.; Chuev, G. N.; Cramer, C. J.; Daily, J.; Deegan, M. J. O.; Dunning, T. H.; Dupuis, M.; Dyall, K. G.; Fann, G. I.; Fischer, S. A.; Fonari, A.; Früchtl, H.; Gagliardi, L.; Garza, J.; Gawande, N.; Ghosh, S.; Glaesemann, K.; Götz, A. W.; Hammond, J.; Helms, V.; Hermes, E. D.; Hirao, K.; Hirata, S.; Jacquelin, M.; Jensen, L.; Johnson, B. G.; Jónsson, H.; Kendall, R. A.; Klemm, M.; Kobayashi, R.; Konkov, V.; Krishnamoorthy, S.; Krishnan, M.; Lin, Z.; Lins, R. D.; Littlefield, R. J.; Logsdail, A. J.; Lopata, K.; Ma, W.; Marenich, A. V.; Martin del Campo, J.; Mejia-Rodriguez, D.; Moore, J. E.; Mullin, J. M.; Nakajima, T.; Nascimento, D. R.; Nichols, J. A.; Nichols, P. J.; Nieplocha, J.; Otero-de-la Roza, A.; Palmer, B.; Panyala, A.; Pirojsirikul, T.; Peng, B.; Peverati, R.; Pittner, J.; Pollack, L.; Richard, R. M.; Sadayappan, P.; Schatz, G. C.; Shelton, W. A.; Silverstein, D. W.; Smith, D. M. A.; Soares, T. A.; Song, D.; Swart, M.; Taylor, H. L.; Thomas, G. S.; Tipparaju, V.; Truhlar, D. G.; Tsemekhman, K.; Van Voorhis, T.; Vázquez-Mayagoitia, Á.; Verma, P.; Villa, O.; Vishnu, A.; Vogiatzis, K. D.; Wang, D.; Weare, J. H.; Williamson, M. J.; Windus, T. L.; Woliński, K.; Wong, A. T.; Wu, Q.; Yang, C.; Yu, Q.; Zacharias, M.; Zhang, Z.; Zhao, Y.; Harrison, R. J. NWChem: Past, present, and future. *The Journal of Chemical Physics* **2020**, *152*, 184102.

(S3) Chai, J.-D.; Head-Gordon, M. Systematic optimization of long-range corrected hybrid density functionals. *J. Chem. Phys.* **2008**, *128*, 084106.

(S4) Chai, J.-D.; Head-Gordon, M. Long-range corrected hybrid density functionals with damped atom–atom dispersion corrections. *Phys. Chem. Chem. Phys.* **2008**, *10*, 6615–6620.

(S5) Lin, Y.-S.; Li, G.-D.; Mao, S.-P.; Chai, J.-D. Long-Range Corrected Hybrid Density

Functionals with Improved Dispersion Corrections. *J. Chem. Theory Comput.* **2013**, *9*, 263–272.

(S6) Grimme, S.; Antony, J.; Ehrlich, S.; Krieg, H. A consistent and accurate *ab initio* parametrization of density functional dispersion correction (DFT-D) for the 94 elements H-Pu. *J. Chem. Phys.* **2010**, *132*, 154104.

(S7) Grimme, S.; Ehrlich, S.; Goerigk, L. Effect of the Damping Function in Dispersion Corrected Density Functional Theory. *J. Comput. Chem.* **2011**, *32*, 1456–1465.

(S8) Grimme, S. Semiempirical GGA-type density functional constructed with a long-range dispersion correction. *J. Comput. Chem.* **2006**, *27*, 1787–1799.
